# Response to Letter to the Editor from Liu et al: “Evidence in Support for the Progressive Nature of Ovarian Endometriomas”

**DOI:** 10.1210/clinem/dgaa542

**Published:** 2020-08-15

**Authors:** Xishi Liu, Giuseppe Benagiano, Ding Ding, Sun-Wei Guo

**Affiliations:** 1 Shanghai OB/GYN Hospital, Fudan University, Shanghai, China; 2 Shanghai Key Laboratory of Female Reproductive Endocrine-Related Diseases, Fudan University, Shanghai, China; 3 Faculty of Medicine and Dentistry, Sapienza, University of Rome, Rome, Italy

We believe that the points raised by Dr Khan and his associates (hereafter, K.F.K.K.) deserve careful consideration. Therefore, we will try to respond to each of their points below.

K.K.F.K.’s first point was based on the marginally significant correlation (*P* = 0.056) between lesional fibrosis and the duration of dysmenorrhea. Given this *P*-value, they believe that our study failed to provide a full evaluation of peritoneal lesions and adhesion. Hence, they assert that “the expression of NK1R and ADRB2 only in OE is overestimated.” We beg to disagree for several reasons. First, the staining was performed objectively, and it could have by no means been “overestimated.” Second, the revised classification of the American Society for Reproductive Medicine classification, which we used, did include the assessment of peritoneal lesions and adhesion, if any. Since it was a study on ovarian endometrioma (OE) progression, our histological analyses were obligatorily confined to OE lesions. If the histologic findings on OE lesions alone are still revealing without an in-depth evaluation of peritoneal lesions, this is all the more cogent. Lastly, the dogmatic insistence on a “magic” threshold (*P* < 0.05) goes against the prevailing opinion of a vast majority of statisticians (1), since it entails the risk of discarding crucial findings.

K.F.K.K.’s second point dealt with the progression of lesional angiogenesis and coloration that “truly indicate the progression phenotype of OE overtime [sic],” implying that our use of markers of epithelial-mesenchymal transition, fibroblast-to-myofibroblast transdifferentiation, smooth muscle metaplasia, and fibrosis were inadequate. This view seems to be based narrowly on their own previous work, but unfortunately overlooks the evidence that these markers can faithfully date endometriotic progression in mice (2) and baboons (3). In addition, lesional fibrosis correlates well with coloration (4) and, as lesions progress and become more fibrotic, lesional vascularity decreases (5), possibly as a result of endothelial-mesenchymal transition (6). In other words, the lesional angiogenesis and coloration can be underpinned by inherent and evolving molecular processes, such as epithelial-mesenchymal transition, fibroblast-to-myofibroblast transdifferentiation, smooth muscle metaplasia, and fibrogenesis. This is especially true since there is no documentation for the direct relationship between angiogenesis and progression, and since the determination of lesion colors is more an art than science. This is all the more important since the extent of lesional fibrosis, a proxy for progression, could be determined by elastography without laparoscopy (7).

With a palpable skeptical overtone, K.F.K.K. lamented that, in the last decade in their hospital, they had not seen any OE case under the age of 20 without an obstruction anomaly. In fact, K.F.K.K.’s hospital performed 350 endometrioma-indicated laparoscopic surgeries between 1982 and 2008 (8), averaging 13.5 cases per year. In our hospital, which is equipped with 20 operating rooms dedicated exclusively to gynecological operations, we perform ~ 2000 endometrioma-indicated laparoscopic surgeries annually, and the 24 adolescent endometrioma cases without any obstructive anomaly were recruited over a time span of ~ 5 years, yielding an estimated prevalence of 0.24% (P = 0.0024). In order to have a reasonably good chance, say, 80%, to encounter at least 1 such adolescent case after performing *n* endometrioma-indicated surgeries, that is *P* (encounter at least 1 such case after performing *n* laparoscopies) ≥0.80, *n* has to be no smaller than log(1-0.80)/log(1-p) = 670. Consequently, if we assume the same amount of case-flow and surgery, it would take Dr Khan and his collegues exactly another four decades in order to have a good chance to encounter at least one such case.

In addition, a review of the literature shows that adolescent OE in subjects without obstructive anomalies is not as rare as they seem to believe (9).

Whether endometriosis is a progressive disease is still a highly contested issue, akin to the Gordian knot. There may be various ways to untie the knot, but the most effective one appears to be the characterization of the molecular processes underlying lesional progression—though granted, lesional progression occurs not only in OE lesions, but also in extraovarian lesions. Yet, to see whether OE progresses or not, one still has to come back to the OE lesion itself, one way or the other.

## Additional Information

***Disclosure Summary:*** The authors have nothing to disclose.
